# Usability and perceptions of a one-on-one telementoring program for young physicians in rural settings of Peru: a mixed method study

**DOI:** 10.1186/s12909-023-04142-2

**Published:** 2023-03-22

**Authors:** Leonardo Rojas-Mezarina, Stefan Escobar-Agreda, Max Chahuara-Rojas, Javier Silva-Valencia, Daniel Hector Espinoza-Herrera, C. Mahony Reátegui-Rivera, Miguel Moscoso-Porras, Juan Quispe-Gamarra, Gerardo Ronceros

**Affiliations:** 1 Unidad de Telesalud, Facultad de Medicina, Universidad Nacional Mayor de San Marcos, Av. Grau 755, Cercado de Lima, 15001 Peru; 2 Asociación Para El Desarrollo de La Investigación Estudiantil en Ciencias de La Salud (ADIECS), Jr. Sergio Bernales 771, Cercado de Lima, 15001 Peru; 3 Escuela de Medicina Humana, Universidad Continental, Av. San Carlos 1980, Huancayo, 12001 Peru

**Keywords:** Medical education, Telementoring, Rural health

## Abstract

**Background:**

Telementoring seems to be a promising strategy to deliver training and counselling to physicians in remote areas. In Peru, early graduated physicians must work for the Rural and Urban-Edge Health Service Program where they face important training needs. The aim of this study was to describe the usage of a one-on-one telementoring program for rural physicians and evaluate the aspects related to the perceptions of acceptability and usability.

**Methods:**

Mixed methods study on recently graduated physicians who work in rural areas and participate in the telementoring program. The program used a mobile application to connect these young doctors with specialized mentors to answer queries about real-life problems raised by working in a rural area. We summarize administrative data to assess participant characteristics and their participation in the program. Additionally, we conducted in-depth interviews to explore the perceived usability, ease of use, and reason for non-use of the telementoring program.

**Results:**

Of 74 physicians (mean age 25, 51.4% women) enrolled, 12 (16.2%) actively used the program and performed a total of 27 queries, which received response in an average time of 5.4 ± 6.3 h. In the interviews, the main reasons for non-use were connectivity issues, feelings of shame, and self-efficacy. For those who used the telementoring program they referred it was easy to use and solve their inquiries timely.

**Conclusions:**

The implementation of a telementoring program sought to provide guidance to recently graduated physicians working in rural areas. Low use rates show that administrative and process-related deficiencies in the program implementation need to be improved.

## Background

Educational interventions for health professionals have been continuously improved with the use of Information and Communication Technologies (ICT) since they facilitate the exchange of information and interaction between educators and their students [1]. However, the implementation of this interventions in rural and remote geographical areas continues to be a challenge, not only due to technical aspects such as connectivity, but also due to perception and usability problems by the health personnel who work there.

One type of these interventions in rural areas is called telementoring, which consists of continuously providing, using ICT, practical and theoretical content from more experienced professionals (mentors) to other professionals with little experience (mentees), focusing on topics of interest depending on the context where the participants are [2]. Although to date it has been shown that it could effectively improve the knowledge, skills and attitudes of health personnel [3, 4], most telementoring experiences follows the ECHO model, so they are usually focused on group sessions [5] and may not have the same applicability for all users. Especially for those groups whose individual needs are very different from each other, the acceptability and usability of the program could be different and a more personalized type of telementoring could be necessary. Therefore, one-on-one telementoring initiatives (the interaction between one mentor and one mentee) have been developed that have shown promising results in capacity building for health human resources, mostly related to medical procediments and surgery topics [6, 7].

In Peru, there is the Rural and Urban-Edge Health Service (SERUMS, in Spanish), where newly graduated physicians (SERUMS physicians) work for a year in primary health care facilities in different rural areas [8]. This service is mandatory since it is a requirement to opt for the second specialty or to work in public health centers. More importantly, it has been seen that these SERUMS physicians face great challenges due to various factors such as lack of experience, training background directed to hospital settings rather than primary-care, not interacting with colleagues, holding new administrative positions, among others [9–11].

The gap in knowledge and expertise that physicians present during the SERUMS is significant, likewise, the training needs would be different according to each case since they could differ, for example, according to their level of previous experience, the health needs of their community, the number of resources that have in their establishment, and the number of responsibilities that they will assume (administrative, clinical, legal, etc.). In this context, the National University of San Marcos (UNMSM, in Spanish) created a one-on-one telementoring program to provide SERUMS physicians with guidance and personalized educational advice during their year of work in remote areas. To carry out this program, a mobile application called JOIN^©^ was used, which is a medical mobile application approved by the FDA to improve communication between health professionals, allowing the delivery of encrypted information, images, among other functionalities such as multi-device support, instant messaging, and video calling.

Since the introduction of new technologies to provide distance education implies a continuous interaction of users with new platforms and applications, these must be evaluated in practice from the perspective of the user (e.g. acceptability and usability) [12, 13]. Then, considering that this program is a first experience of one-on-one telementoring intervention in rural areas of Peru, our objective is to describe the use of the intervention and evaluate the aspects related to the perceptions of acceptability and its usability.

## Methods

### Study design and context

We conducted a mixed-methods study using a sequential explanatory design, which is a recommended approach that sequentially integrates quantitative and qualitative data for evaluating telehealth interventions [14]. First, we analyzed quantitative data from the administrative database of the 2018 cohort of the “UNMSM telementoring program” to describe users characteristics and mobile application usage. Then, from 2019 July to 2019 August, we performed interviews to gather qualitative information regarding usability and perceptions of the program from those physicians who were part of the 2018 cohort.

### Population and sampling

In March 2018, two months prior to SERUMS beginning, 124 physicians were invited to participate in the one-on-one telementoring program by sending emails and telephone contact. Only 74 physicians agreed to participate, the reasons for declining from those who did not accept were not available in UNMSM records. For the quantitative phase of the study, we include data from all physicians who agreed to participate in the program. For the qualitative phase, 16 of those enrolled were interviewed, 8 of them used the program and 8 did not. Said sample size was obtained using the saturation technique, recruiting participants until their answers no longer contributed to the objectives of the interview.

### About the telementoring program

#### Purpose

Because medical training in Peru is hospital-based and often physicians go through many challenges to carry out their professional activities during SERUMS in rural areas (clinical, community, administrative, legal, ethical activities, among others.), a telementoring Program using a mobile application was designed to communicate specialist physicians (mentors) with these newly graduated physicians in rural areas to provide them, in a short period of time, with information and personalized guidance on the problems they consulted.

#### Telementoring app

Within the processes of the telementoring program, a mobile application called JOIN^©^ (Allm Inc., Japan) was used. This was a smartphone application approved in 2015 for FDA that allows healthcare professionals to share clinical data and multiple format files including DICOM medical images.

JOIN© was used as the platform of communication between SERUMS physicians and mentors or technical team. All the information shared via JOIN^©^ follows the international standards HIPAA Compliance for securing confidentiality and anonymity [15]. Participants could share messages and ask questions in group chats, in personal chats and send images, videos, among others. Physicians interested in participating in the telementoring program were required to have a smartphone with an operating system of at least Android 5.0 or iOS 8.0 and able to access the Internet for at least one hour during the day.

#### Users and structure of the telementoring program

**Fig. 1:**
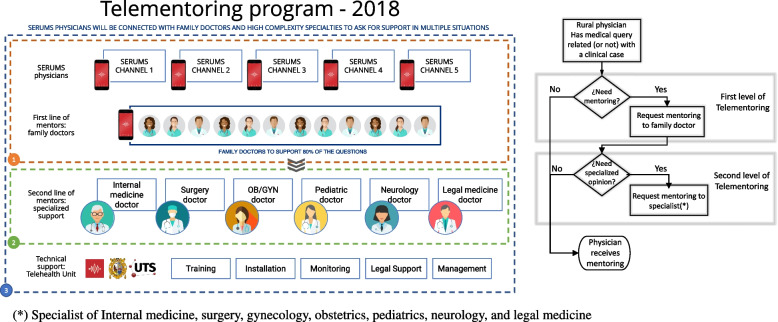
Structure and flow of UNMSM telementoring program. Perú – 2018

The program was structured considering four types of users:

1. Young physicians in rural service, who were the end-users of the program. When the telementoring program started, all enrolled physicians were registered in an instant messaging channel in JOIN^©^, where they could make inquiries by writing in the chat, sending documents, images, videos or in some cases by contacting the mentor with a video call.
2. Mentors from Family medicine. This group was the first line of response to queries made by SERUMS physicians. It was made up of 15 family doctors from Lima (the capital city of Peru), who, based on a schedule, entered JOIN^©^ and answered questions or problems of daily professional practice (clinical, community, administrative, legal, ethical activities, among others).
3. Mentors from other specialties. This was the second line of response when the family doctors considered that the consultations required the opinion of another specialist doctor. The program had one specialist from each of the following medical areas: internal medicine, surgery, gynecology, obstetrics, pediatrics, neurology, and legal medicine
4. Technical team. They provided technical support and ensured proper communication between SERUMS physicians and mentors when a query arose. It was made up of physicians, communications professionals, and IT professionals. The structure and workflow can be seen in Fig. 1.

#### Implementation

Recruitment: Graduates of 2018 from the UNMSM School of Medicine who would start SERUMS were invited to an informational meeting about the telementoring program. The invitation was made through the institutional social media and website. Everyone who attended the informational meeting was then asked to register for the program through an online form.

Training: Before starting the program, physicians and mentors received two weeks of training on the use of the application. This was done through asynchronous online training using a virtual classroom on the open platform Moodle. For this, multimedia materials (videos and documents) were used to teach about the use of the program including login, creation of user accounts, sending text, images, communication with mentors and video calls.

Program engagement and monitoring: The use of the telementoring program was not mandatory. The participants could use the application if they consider that they need guidance with a situation in their professional work.

Legal aspects and confidentiality of information: All participants had to sign a document of the terms and conditions of participation, where it was mentioned that the program was designed to provide personalized educational advice, not to solve clinical cases and it should not be used for emergency situations. The program does not represent a remote medical work, for which it was prohibited to carry out consultations sharing personal information of patients. The terms and conditions of use of this telementoring program can be downloaded in link [16].

Program length: the program was offered from June 2018 to December 2018

Program funding: The program was financed by UNMSM. The license to use the JOIN^©^ application was financed by the Ministry of Telecommunications of Japan and granted to the UNMSM for research projects during the year 2018–2019.

### Measures and data analysis

First, we collected quantitative data from SERUMS physicians who enrolled in the telementoring Program in 2018 available in UNMSM registries of the telementoring program. Some variables were the level of resolution of the healthcare facility they work according to Peruvian normativity [17], dependency, quintile of poverty, and location. In addition, we collected data regarding mobile application usage, such as the total number of queries, attended cases, average response-time and medical specialties required from the JOIN^©^ app records. Descriptive analyses were performed using Stata 16 (Stata Corp, College Station, TX, USA). Numerical variables were summarized means and standard deviations, and categorical variables were summarized as frequencies and percentages.

Then, we collected qualitative information through telephone interviews. We explored perceived ease of use, usefulness, and reasons for participating in the telementoring program. The interviews lasted an average of 15 min and were audio recorded. For the analysis, the ATLAS.ti v7.0 software was used, applying the thematic analysis methodology [18]. Content was first coded from text segments that were related to the research questions. Subsequently, these codes were linked to generate categories that outline the perceptions of SERUMS physicians.

## Results

### Usage

Of 74 SERUMS physicians who agreed to participate in the telementoring program and completed the initial training, 38 (51.4%) were women and more than 70% of the participants reported no previous experience with medical applications and that 79.5% reported that they worked in health facilities located in areas of poverty and extreme poverty that depended mainly on the Ministry of Health of Peru (See Table 1).

**Table 1:** Characteristics of the SERUMS physicians enrolled and their healthcare facilities. Perú 2018

Characteristics	*N* = 74
	**n (%)**
**Gender**	
Female	38 (51.4)
Male	36 (48.7)
**Previous use of medical apps**	
No	52 (70.3)
Yes	15 (20.3)
Does not respond	7 (9.5)
**Poverty quintile**	
Quintile 1 (poorest)	58 (79.5)
Quintile 2	13 (17.8)
Quintile 3	2 (2.7)
**Resolution Level of the H.F**	
I-1	15 (20.6)
I-2	44 (60.3)
I-3	14 (19.2)
**Dependency of the H.F**	
Ministry of Health	55 (75.3)
Social security (EsSalud)	13 (17.8)
National Police Department	2 (2.7)
National Armed Forces	3 (4.1)
**Geographic location of the H.F**	
Coastal regions	10 (13.7)
Highland regions	59 (80.9)
Jungle regions	4 (5.4)

*H.F* Healthcare Facility

Only 12 (16.2%) of physicians enrolled used the program, that is, made inquiries or questions to the mentors. A total of 27 consultations were carried out, with a mean of 2.1 ± 1.3 consultations for each physician. All queries were answered. The majority (62.5%) were answered in less than six hours and were mainly related to the topics of dermatology (40.7%), legal medicine (14.8%) and cardiology (11.1%) (See Table 2).

**Table 2:** Characteristics of the utilization of the telementoring program on young physicians in rural areas. Perú 2018

Characteristics	*N* = 27
	**n (%)**
**Average of response time (hours)**	5.4 ± 6.3
**Mentoring areas**	
Dermatology	11 (40.7)
Legal Medicine	4 (14.8)
Cardiology	3 (11.1)
Pediatrics	2 (7.4)
Endocrinology	1 (3.7)
Infectology	1 (3.7)
Neonatology	1 (3.7)
Neurology	1 (3.7)
Ophthalmology	1 (3.7)
Otorhinolaryngology	1 (3.7)
**Response time**	
< 6 h	15 (62.5)
6–12 h	3 (12.5)
> 12 h	6 (25.0)
**Average queries by physician**	2.1 ± 1.3

**Fig. 2:**
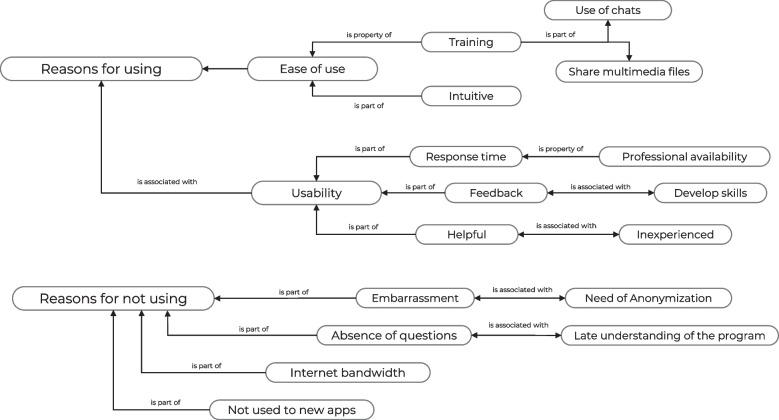
Reasons for using and not using a telementoring Program based on a mobile application for young physicians in rural areas. Categories obtained from the content analysis of the interviews

Regarding the interviews carried out, we explored the ease of use and usability of the system among physicians who used the telementoring program, and we explored the main reasons for not using the system among those who did not use the system during the study period (See Fig. 2).

### Easy to use

SERUMS physicians who used the program reported that was easy to use because it was intuitive and similar to other instant messaging systems, they were more familiar with, such as WhatsApp groups. Also, due to the possibility of sharing a specific consultation in a structured way by attaching multimedia files, which were necessary for consultations with some specialties such as dermatology or pediatrics.

*“I think it is a good tool to help us in our medical work. Especially for some clinical issues since in the group chat, we could share some clinical data in a structured way and images in good resolution.” (Participant 7).*

However, it was also reported that some physicians found it difficult to use other features such as starting a personal chat, notification settings, etc. For this, the need for additional training was raised due to the little previous experience of some physicians in the use of medical applications.

*“Almost all individual questions were done in the group chat. I didn’t really know how to use personal chat because it wasn’t very clear to me in the initial training and I’m not very familiar with apps. On certain occasions I would have liked to ask questions in private.” (Participant 1).*

### Usability

It was reported that the system was of great help in resolving cases since, due to the physicians’ lack of experience, they were not clear on how to act in certain situations. Also, because the queries of others could be seen in the chat, and served as learning material to develop skills to solve similar problem situations.

*“It has been an important and useful tool because in some specific issues we had inexperience. Especially since we are working at the first level of care, and we do not receive specific training for some situations here… It was also useful when you share and see the cases of your colleagues, it encourages you to think about similar problems, you learn from it.” (Participant 4) .*

Additionally, it was mentioned that the response time of the questions played an important role. Although they initially expected responses to be immediate, given the nature of the program (not used for urgent/emergencies) and given the limited availability of specialists, most agreed that the time delay was adequate.

*“When I joined the program, I was inexperienced, so I was encouraged to consult ... now I have more knowledge in these aspects, for example, how to act when faced with an autopsy request for a legal medical problem ... The program it is especially useful in the first months of SERUMS.” (Participant 10).*

### Reasons for not using the system

It was reported that although it would have been very helpful to have the opinion of a specialist physicians or a university professor, recent graduated physicians did not ask questions because they had a certain degree of embarrassment and fear of appearing foolish or inexperienced to other colleagues. It was reported that they would prefer no group chats, only direct communications, and that it would be good for some not to show their full name but an alias.

*“It seemed that those who did write in the groups put really difficult cases… and it was embarrassing to write simple questions. I know of a friend who sometimes had questions but didn’t want to show that he didn’t know because they could later identify him with his full name. In the case of us (physicians)... even more so, we are too proud to admit it.” (Participant 2).*

Another reason was because they started accessing the program late when they had already obtained enough experience to solve most of the frequent doubts on their own. The reason for this was that they had never participated in a similar telehealth program before and did not fully understand how it worked. Most agreed that the program should be prioritized in the first months of social service.

*“In my case at this point I have no longer had any doubts about cases. That would be the main reason why I did not have the need to consult or present a clinical case. It would have been especially helpful at the beginning of SERUMS when we were adapting”. (Participant 4).*

Other reasons referred by SERUMS physicians were related to technical aspects. Internet bandwidth was mentioned to be a main issue, being in most cases of poor quality or simply inaccessible for some settings. Also, some physicians mentioned they did not desire to install new mobile-phone apps, instead they preferred and use frequently commercial messaging apps, such as WhatsApp, to performed clinical queries to their peers.

*“I do not use the application because of the internet problem… The truth is that the connection was bad almost all last year, only calls or text messages came in” (Participant 13)*.

## Discussion

### Principal results

Our results reflect that the participation rates in this first version of the telementoring program were low and that the physicians indicated the feelings of embarrassment to ask in groups, the lack of familiarity with new apps and low connectivity as the main reasons. However, users who did use the system reported that the program was easy to use and useful for resolving their queries in a timely manner.

Other one-on-one telementoring experiences showed to be equally effective on developing medical procediments compared to physical mentoring or virtual reality assisted training [6, 7, 19, 20]. Nonetheless, most one-on-one telementoring reports has the aim of developing particular prodecimental skills and were oriented to surgical settings, so its application on primary health and most diverse set of topics still uncertain. Also, this type of telementoring strategy only covers the lack of knowledge of health personnel in the context of predefined medical topics and not in complex real-life situations. In contrast, the telementoring program studied in this research was designed as an on-demand telementoring service to support the specific medical queries of each SERUMS physicians through responses from specialist physicians using a new medical mobile application.

In general, all mentoring programs have the advantage of being more student-centered than traditional teaching [21]. However, despite this, which theoretically also promotes student motivation and engagement [22], our findings in this experience revealed low usage rates that could be due to several reasons.

First, the design of the program did not include conducting an educational survey to assess the specific needs of graduate physicians and how they would like to be provided with such information. Consequently, despite previous evidence suggesting an important need for clinical guidance during SERUMS [23], the main needs of medical graduates could actually be different. Some of these needs could include aspects of health management, legal medicine, ethics, or public health, so it would be necessary to include experts in these topics within the mentoring. This is especially relevant since most of their training at the university is mainly oriented to clinical aspects and not to these other activities [24].

Second, some SERUMS physicians referred to the presence of limited mobile internet connectivity in their healthcare facilities. This is a common problem in rural areas, where regardless of mobile phone signal coverage, connectivity could be affected considering various geographic and climatic conditions [25]. Despite this, since the program was not geared towards emergency/urgency cases and immediate responses were not necessary, we do not consider this to be a limiting factor for implementation. Due to this characteristic, if necessary, the use of voice calls or text messages could be included, which require less technical infrastructure for their application in rural areas [26].

Thirdly, despite having a telementoring system with family doctors and specialist doctors available, the SERUMS physicians preferred to consult with their own networks of contacts, generally made up of other recently graduated physicians, with whom they had more confidence. Some degree of embarrassment in asking questions and fear of appearing inexperienced to other colleagues was reported. This situation was also evidenced in other countries such as Colombia [27] and suggests that future telementoring interventions for recently graduated physicians should include the presence of previously known professionals, such as university professors of medicine and with experience who provide greater confidence. In addition to enabling ways to ask questions privately or anonymously. Another alternative would be to include a group telementoring approach, which has shown evidence of improving knowledge, perceived ability and patient outcomes in primary care [28, 29] and/or rural settings [27, 30, 31]; and which also has the advantage of reducing health professionals reluctance to participate, as they report feeling integrated into their learning teams [28].

Fourth, most of the physicians who participated in the study had no prior experience using medical apps. This would imply that, despite having a smartphone, it is possible that many of them do not trust or prefer not to use new applications or technological tools to support their professional training, as observed in medical students and recent graduates in our country [32, 33].

On the other hand, among the physicians who did actively use the Telementoring program, it was found that most of the consultations are related to dermatology and legal medicine, specialties not initially included in the program. Possibly due to little prior training in these specialties at the primary care level during university [34]. This leads us to think that it is important to include more specialists from different branches in a telementoring program for primary care, so it is important to explore the needs of users before implementing it [35].

In terms of ease of use, our findings were similar to a study conducted at Jikei University Hospital in Japan, in which interns reported that the use of the JOIN© was intuitive and similar to other apps. Messaging as LINE [36]. Still, the need for additional training was also raised. This could be explained by their lack of previous experience with the use of medical applications, which was reported by almost 70% of the physicians enrolled in the program. Although this is contradictory with other Peruvian studies, where they show that 97% of the interviewees reported that they used some medical application and 72% used applications to share medical images or videos [37]. Another explanation could be due to the fact that the training provided was carried out through an asynchronous and unsupervised online course, which, although it allowed progress at the student’s pace and to review the materials at any time, could not have been completed correctly. This is consistent with evaluations of a massive open online courses that showed a dropout rate of 84.5% of registered participants, regardless of geographic location [38].

### Limitations and strengths

Although these are important findings, some limitations must be considered. First, the recruitment of participants was not random but was done through invitations by mail and telephone, which implies a possible selection bias, more specifically a voluntary bias. Second, the interviews were conducted at the end of the SERUMS year, so there might be some possibility of recall bias. Finally, we do not know if the sociodemographic or occupational characteristics of physicians from other universities are similar to those from UNMSM, so our findings may not be extrapolated to all contexts.

Despite these limitations, our study provides important insights into the potential of telementoring interventions using mobile technologies to close knowledge gaps in rural physicians in low- and middle-income countries. Although we were unable to measure the direct impact of the program, our results suggest that this impact exists and would merit evaluation in future cohorts. In addition, the Telementoring program presented several strengths that have previously been shown to generate positive results in medical education, such as the ease of use of the technology, the confidential transmission of information, and the adequate response time to queries about real problem situations of a recently graduated physicians working in a rural area [39].

## Conclusions

Telementoring through a mobile application represents a useful alternative to provide educational support to young physicians who work in rural areas and who need help to face problems that arise in their professional work. However, its implementation may be limited by the physicians' lack of experience in using mobile applications, limited Internet access, lack of anonymization when asking questions, and the presence of medical specialist mentors with whom they did not trust to ask openly. This tells us that telementoring interventions designed for physicians working in rural areas should include the considerations discussed above such as conducting an initial formative study and a final evaluation of the impact of improving physicians' experience while serving in rural areas.

## Data Availability

The Faculty of Medicine of the UNMSM has possession of the raw quantitative information regarding the Telementoring program and reserves the right to share it publicly, but it can be requested for research purposes through a formal request to the faculty at telesalud.fm@unmsm.edu.pe. Interview respondents were assured raw qualitative data would remain confidential and would not be shared, hence, data are not available, but are available from the corresponding author upon request.

## Abbreviations

SERUMS: Rural and Urban-Edge Health Service Program
UNMSM: National University of San Marcos
FDA: Food and Drug Administration
ICT: Information and Communication Technology
